# Oxidation-Tuned CuO_*x*_ for Spin–Orbit Torque Efficiency Enhancement

**DOI:** 10.1021/acsami.5c15854

**Published:** 2025-10-21

**Authors:** Che-Jui Li, Chi-Feng Pai

**Affiliations:** Department of Materials Science and Engineering, 33561 National Taiwan University, Taipei 10617, Taiwan

**Keywords:** Spin−orbit torque, orbital Hall effect, orbital Rashba−Edelstein effect, orbital-to-spin conversion, harmonic Hall voltage measurement

## Abstract

In this study, we demonstrate that oxidation-controlled CuO_*x*_ layers can serve as effective sources of orbital current for enhancing damping-like spin–orbit torque (SOT) in CoFeB/Pt heterostructures. By reactively sputtering CuO_*x*_ under varied oxygen concentrations, we systematically tuned its oxidation state and evaluated its impact on SOT efficiency through harmonic Hall voltage measurements. A peak damping-like SOT efficiency of |ξ_*DL*_| ≈ 0.30 was achieved at Q = 4% with a CuO_*x*_ thickness of 3 nm and a Pt thickness of 4 nm, representing a ∼76% enhancement over the Pt-only control structure. SOT efficiency exhibited a nonmonotonic dependence on CuOx thickness, peaking at 3 m and then decreasing, suggesting limited propagation or interfacial conversion saturation. Similarly, tuning Pt thickness revealed that orbital-to-spin conversion is most effective around 4 nm, consistent with the expected spin diffusion behavior. In contrast, naturally oxidized Cu samples reached a maximum |ξ_*DL*_| of ≈0.23 (∼35% enhancement), with limited tunability. These results confirm that while both natural and reactive oxidation can induce orbital torques, only controlled reactive sputtering yields consistently strong and stable effects. The findings establish CuO_*x*_ as a scalable orbital torque source and underscore the importance of oxidation control and interfacial engineering in next-generation spintronic devices.

## Graphical abstract

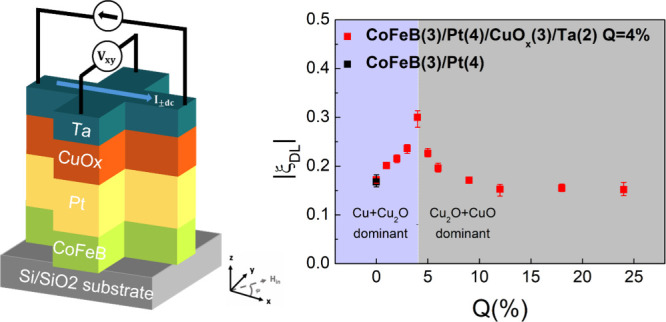

## Introduction

Spin–orbit torque (SOT) allows electrical control of magnetization, underpinning key advances in modern spintronic technologies. SOT-driven switching in magnetic memory devices like SOT-MRAM offers fast operation, low write energy, and high endurance.^1–3^ These advantages make SOT-based devices attractive for embedded computing, AI accelerators, and neuromorphic architectures where efficient, nonvolatile magnetization control is essential. SOT arises when a charge current in a heavy metal generates a transverse spin current that transfers angular momentum to an adjacent ferromagnetic layer, where angular momentum transfer exerts torque on the magnetization.^4,5^ This spin current is typically produced by the spin Hall effect^6^ (SHE) in heavy metals such as Pt,^7^ Ta,^8^ or W,^9^ or by spin accumulation at asymmetric interfaces via the spin Rashba-Edelstein effect (SREE).^10,11^

While these mechanisms have enabled practical SOT devices, they face critical limitations. The SHE requires materials with strong spin–orbit coupling, which are often rare, expensive, or exhibit undesirable electrical properties. Moreover, the spin Hall angle, which characterizes the efficiency of charge-to-spin conversion, is often difficult to optimize independently of other parameters such as conductivity.^12,13^ Meanwhile, SREE-driven torques are confined to atomic-scale interfaces and highly sensitive to structural disorder, capping layers, and chemical intermixing, which make their performance hard to tune in practice.

To address these challenges, the emerging field of orbitronics has introduced orbital angular momentum (OAM) as a complementary degree of freedom for generating and manipulating torques.^14,15^ Two key orbital mechanisms have been proposed: the orbital Hall effect (OHE) and the orbital Rashba–Edelstein effect (OREE). The OHE involves a transverse flow of orbital current under an electric field, originating from the orbital texture of Bloch wave functions, even in the absence of strong SOC.^16–18^ In contrast, the OREE is an interfacial mechanism that relies on inversion symmetry breaking and orbital hybridization to induce orbital accumulation at the boundary.^19–21^

Recent theoretical work indicates that many materials can exhibit large orbital Hall conductivities due to their intrinsic band structure.^22,23^ These orbital currents are especially interesting because they are not subject to the same limitations as spin currents in terms of dephasing or short diffusion lengths.^24,25^ Additionally, orbital angular momentum can be converted to spin angular momentum via interfacial spin–orbit coupling or hybridization mechanisms, providing an efficient orbital-to-spin conversion pathway even in systems with weak SOC.^26,27^ These mechanisms offer new strategies for leveraging light-element or semiconducting materials in spintronic systems, expanding the material palette beyond traditional heavy metals.

Among potential orbital current sources, copper oxides (CuO_*x*_) have attracted growing attention. While elemental Cu lacks strong spin–orbit coupling, it exhibits high conductivity and favorable orbital character.^20,28,29^ Upon oxidation, the resulting CuO_*x*_ introduces broken inversion symmetry and modulated orbital structure, making CuO_*x*_ a promising platform for orbital current generation. Prior studies have reported enhanced SOT efficiencies in systems with naturally oxidized Cu or Cu/oxide interfaces, often attributing the effect to interfacial mechanisms such as the orbital Rashba–Edelstein effect (OREE).^21,30–32^

However, natural oxidation is uncontrolled, leading to variability in phase composition and interface quality and limiting reproducibility. In this work, we employ reactive sputtering to precisely tune the Cu oxidation level, synthesizing CuO_*x*_ films from metallic Cu to mixed Cu_2_O/CuO phases, which we integrate into heavy-metal/ferromagnet heterostructures. Harmonic Hall voltage measurements reveal how the CuO_*x*_ oxidation state influences the strength of the damping-like SOT. Our findings demonstrate that oxidation engineering is a powerful tuning knob for enhancing orbital torque performance. This work lays the foundation for future strategies that leverage orbital phenomena in complex oxide systems and demonstrates that Cu-based oxides offer a scalable and versatile platform for spintronic device innovation when properly controlled.

## Experimental Section

### 1 Sample Preparation

All samples were grown on thermally oxidized Si/SiO_2_ substrates using a high-vacuum DC magnetron sputtering system with a base pressure below 2 × 10^–7^ Torr to minimize contamination. Pt, Ta, Cu, and CoFeB were grown by direct current (DC) sputtering with a power of 30 W under an Ar pressure of 3 mTorr. During the deposition process, the substrate holder was rotated at 20 rpm to ensure the film homogeneity. All film thicknesses were calibrated using an alpha-step profilometer.

The primary focus of this study is a series of samples incorporating a CuO_*x*_ layer deposited by reactive sputtering above the Pt layer: CoFeB (3 nm)/Pt (*d*_*Pt*_)/CuO_*x*_ (*d*_*CuOx*_)/Ta (2 nm). The thicknesses of Pt and CuO_*x*_ were independently varied from 1 to 5 nm to examine their influence on spin–orbit torque efficiency. By systematically varying the O_2_/Ar gas ratio during sputtering, we were able to reproducibly tune the oxidation level of the CuO_*x*_ layer. This approach enables precise control over the orbital-related interfacial properties affecting spin–orbit torque generation. The oxidation condition is defined by the parameter Q, calculated as

$$Q=\frac{O_{2}\mathrm{gas}\;\mathrm{flow}\;\mathrm{rate}}{\mathrm{Ar}\;\mathrm{gas}\;\mathrm{flow}\;\mathrm{rate}+O_{2}\mathrm{gas}\;\mathrm{flow}\;\mathrm{rate}}$$ 1.1

where the total gas flow was fixed at 40 sccm. Samples with Q = 0%, 1%, 2%, 3%, 4%, 5%, 6%, 9%, 12%, 18%, and 24% were fabricated to probe the effect of oxidation level on SOT efficiency. Note that varying Q also slightly affects the deposition rate (see Supporting Information 1).

For comparison, two additional sample types were prepared. First, a control sample without any CuO_*x*_ layer: CoFeB (3 nm)/Pt (*d*_*Pt*_), representing the baseline spin Hall-driven structure. Second, a structure with a 3 nm metallic Cu layer inserted above the Pt layer: CoFeB (3 nm)/Pt (*d*_*Pt*_)/Cu* (3 nm). In this sample, the Cu* undergoes natural oxidation upon exposure to air, providing a qualitative contrast to the controlled reactive oxidation sample.

As illustrated in Figure 1, the basic heterostructures studied in this work consist of a CoFeB/Pt bilayer, with or without an additional Cu-based layer. In the control structure, Pt serves as the spin current source, generating transverse spin currents via the spin Hall effect, which are injected into the adjacent CoFeB layer to induce spin–orbit torques. In the modified structure, a CuO_*x*_ (or naturally oxidized Cu*) layer is deposited on top of the Pt layer to serve as a potential orbital current source. In this configuration, orbital angular momentum (L) generated in the CuO_*x*_ layer may be converted into spin angular momentum (S) as it propagates across the CuO_*x*_/Pt interface, providing an additional torque component on the CoFeB layer. The Ta capping layer fully oxidizes upon air exposure, forming a stable TaO_*x*_ layer that acts as a barrier against further oxidation of the underlying CuO_*x*_. Because the oxidized Ta layer is electrically insulating, it does not contribute to charge or spin transport and thus does not affect the SOT efficiency measurements (See Supporting Information 2). After deposition, the multilayers were patterned into Hall bar devices with a channel width of 10 μm using standard UV photolithography and Ar ion milling. Electrical transport and harmonic Hall measurements were performed at room temperature under ambient conditions.

**Figure 1:**
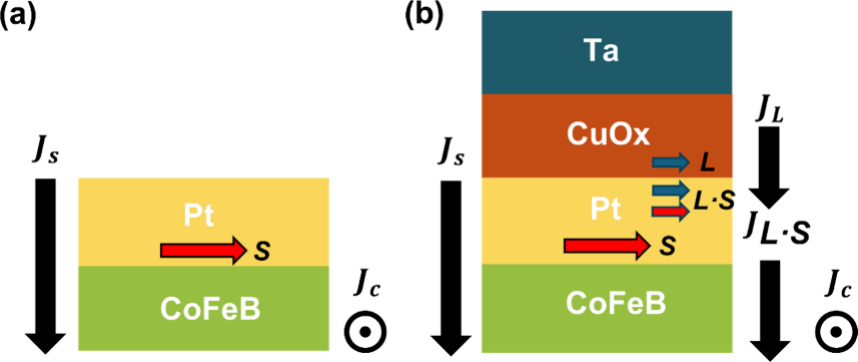
Schematic comparison of spin–orbit torque generation in (a) conventional CoFeB/Pt bilayer and (b) CoFeB/Pt/CuO_*x*_/Ta heterostructure. In (a), spin current is generated via the spin Hall effect in Pt and injected into the ferromagnet. In (b), an additional orbital current is generated in the CuO_*x*_ layer and converted to spin current near the CuO_*x*_/Pt interface via orbital-to-spin conversion, contributing to enhanced damping-like torque. The presence of both spin and orbital channels in the CuO_*x*_-inserted structure enables more efficient spin current injection into the ferromagnet.

### 2 Harmonic Hall Voltage Measurement

We employed harmonic Hall voltage measurements using a DC-based planar Hall effect (PHE) curve shift method to quantitatively probe the current-induced torques in our heterostructures.^33–36^ This technique allows for the extraction of spin–orbit torque components by comparing angular-dependent Hall resistance measured under opposite current directions. For each field angle, the Hall voltage was measured twice: once under + I and once under -I. The components of the first and second harmonic signals were extracted as

$$\begin{array}{c} R_{H}^{1\omega }=\left(R_{H}^{+}+R_{H}^{-}\right)/2\\ R_{H}^{2\omega }=\left(R_{H}^{+}-R_{H}^{-}\right)/2 \end{array}$$ 1.2

The experimental setup is schematically illustrated in Figure 2(a). A Keithley 2400 source meter was used to apply a constant DC current, while the transverse Hall voltage was recorded using a Keithley 2000 multimeter. The sample was placed under a rotating in-plane magnetic field with fixed amplitude generated by a vector electromagnet. The field angle was swept from 0° to 360°, with the angle defined between the current direction and the magnetic field.

**Figure 2:**
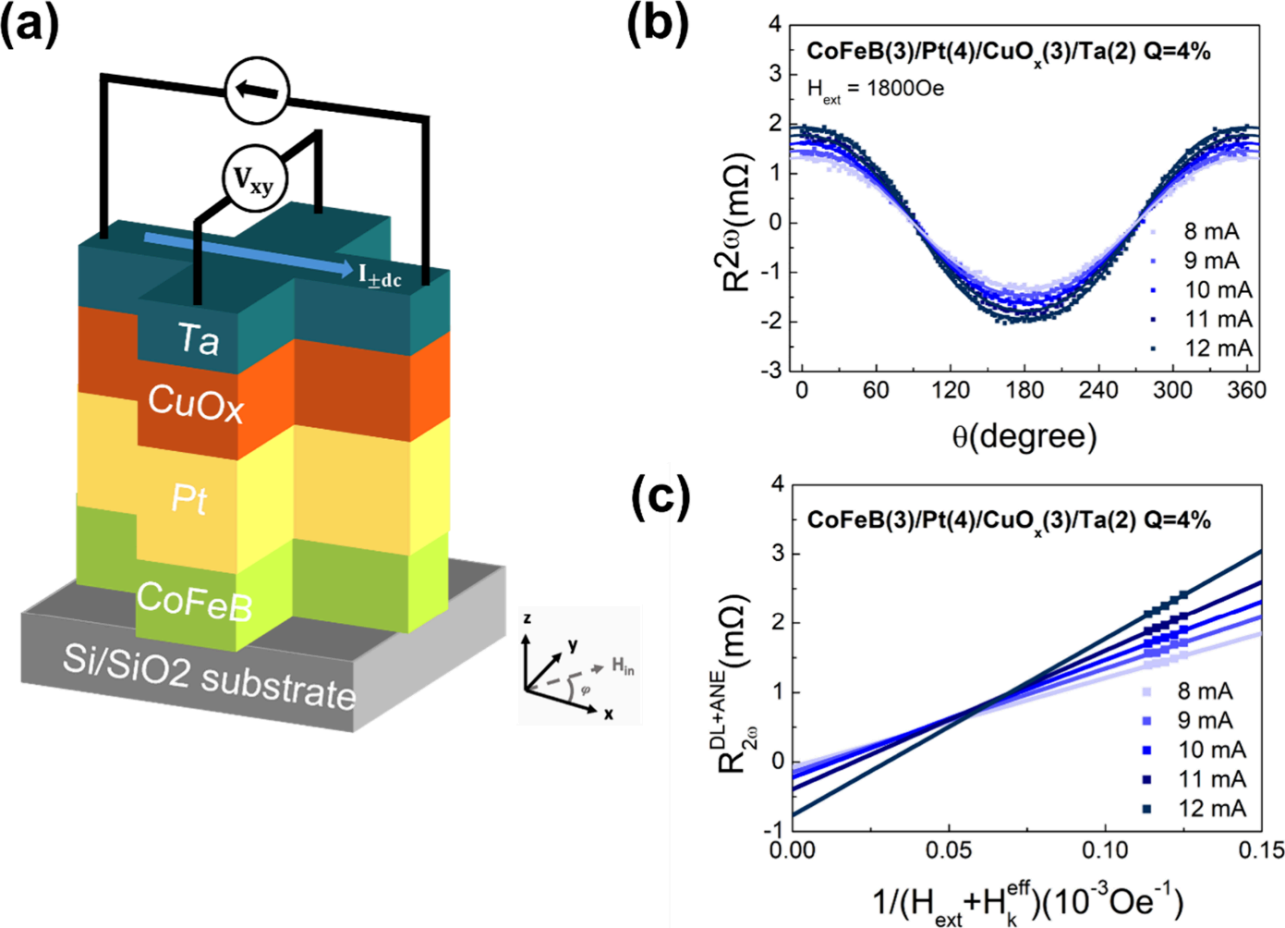
(a) Schematic of the harmonic Hall voltage measurement setup. (b) The *R*_*H*_^2ω^ data as a function of in-plane magnetic field angle under various currents and a fixed magnitude of 1800 Oe. (c) Field dependence fitting of *R*_2ω_^*DL* + *ANE*^under different applied currents.

The *R*_*H*_^2ω^contains contributions from the current-induced effective fields and thermal contributions. The damping-like torque tilts the magnetization into the out-of-plane direction and creates a Hall voltage via the anomalous Hall effect (AHE). The field-like torque tilts the magnetization within the plane, resulting a Hall voltage via the planar Hall effect (PHE). The angular dependence of *R*_*H*_^2ω^ obtained from CoFeB­(3 nm)/Pt­(4 nm)/CuO_*x*_(3 nm)/Ta­(2 nm) with Q = 4% for different applied currents and a fixed *H*_*ext*_ = 1800 Oe are shown in Figure 2(b). The angular dependence of *R*_*H*_^2ω^ can be modeled as

$$R_{H}^{2\omega }=-\left(R_{AHE}\frac{H_{DL}}{H_{ext}+H_{k}^{eff}}+R_{2\omega }^{ANE}\right)\cos\varphi -\left(2R_{PHE}\frac{H_{FL}+H_{Oe}}{H_{ext}}\right)\cos\varphi \cos2\varphi +R_{2\omega }^{PNE}\sin2\varphi =R_{2\omega }^{DL+ANE}\cos\varphi +R_{2\omega }^{FL+Oe}\cos\varphi \cos2\varphi +R_{2\omega }^{PNE}\sin2\varphi$$ 1.3

The first term captures contributions from the damping-like torque and anomalous Nernst effect (ANE), the second arises from the field-like torque and Oersted field, and the third term originates from the planar Nernst effect (PNE). To extract the spin–orbit torque components, measurements were repeated under different magnetic field strengths. In this work, we focus primarily on the analysis of *H*_*DL*_, as *H*_*FL*_ is typically an order of magnitude smaller. The field dependence of the *R*_2ω_^*DL* + *ANE*^ was analyzed using the following equations:

$$R_{2\omega }^{DL+ANE}=-R_{AHE}\cdot H_{DL}\cdot {\left(H_{ext}+H_{k}^{eff}\right)}^{-1}+\left(-R_{2\omega }^{ANE}\right)$$ 1.4

By fitting the field dependence of *R*_2ω_^*DL* + *ANE*^, as shown in Figure 2(c), the slope and intercept of the linear fit allow the separation of the damping-like effective field *H*_*DL*_ and the thermal terms *R*_2ω_^*ANE*^.

#### Q Dependence

The resistivity of the CuO_*x*_ films shows a clear monotonic increase with increasing oxygen content Q. As shown in Figure 3(a), for Q = 0%, corresponding to pure metallic Cu, the resistivity is low (∼10 μΩ·cm), and increases gradually. Beyond Q = 12%, the resistivity rises sharply, exceeding 100 μΩ·cm at Q = 24%. This trend reflects the progressive oxidation of Cu, where low-Q samples remain largely metallic, intermediate-Q films develop semiconducting character due to Cu_2_O formation, and high-Q samples become dominated by insulating CuO. These electrical measurements are corroborated by Cu 2p X-ray photoelectron spectroscopy (XPS). As shown in Figure 3(b), the Cu 2p spectrum shows narrow peaks without shakeup satellites at Q = 0%. The Cu 2p_3_/_2_ peak is around 932.6 eV, which is consistent with metallic Cu.^37^ For Q = 3%, the main peaks broaden slightly, and weak shoulders emerge, indicating the presence of Cu^+^ species associated with Cu_2_O. The Cu 2p_3_/_2_ binding energies for Cu (932.63 eV) and Cu_2_O (932.18 eV) are nearly indistinguishable within typical experimental resolution, and Cu^+^ does not exhibit the distinct satellite peak characteristic of Cu^2+^.^38,39^ In our spectra, the absence of a Cu^2+^ satellite at low Q suggests the presence of reduced states such as Cu_2_O. This interpretation is consistent with some previous studies, which reported the formation of Cu_2_O in the low-oxidation regime of Cu.^30,40^ For Q = 6%, the Cu^2+^ satellite peak begins to appear, indicating that CuO has already formed in the sample. At Q = 12%, prominent Cu^2+^ satellite peaks appear around 940–945 and 962 eV, confirming the predominant presence of CuO. This evolution confirms a compositional transition from Cu → Cu_2_O → CuO with increasing Q.

**Figure 3:**
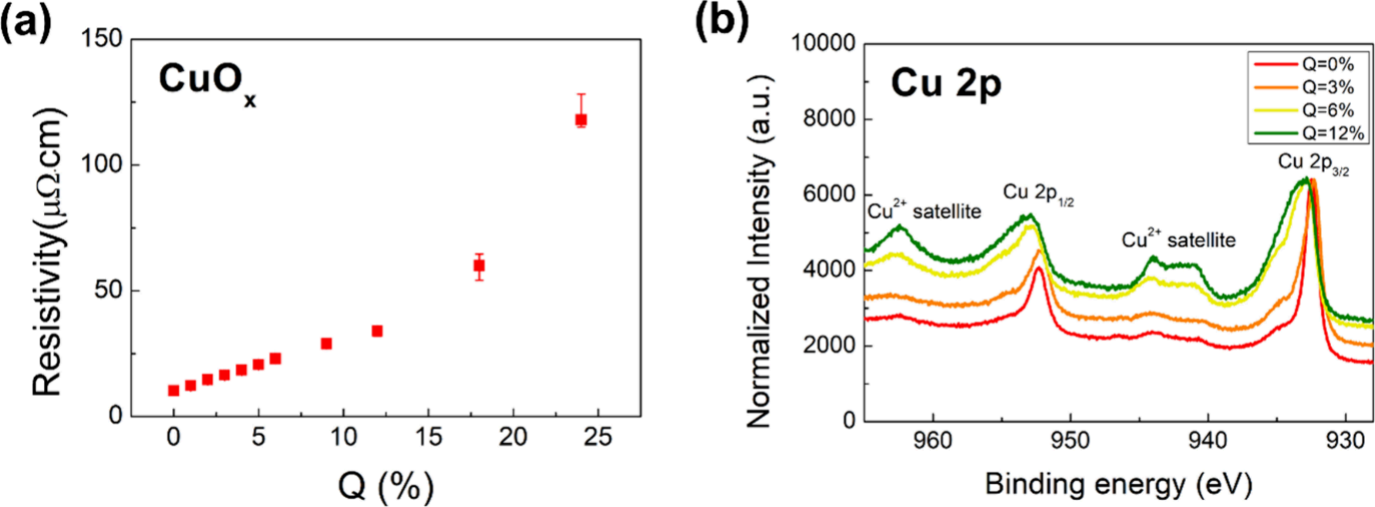
(a) The resistivity of CuO_*x*_ grown under different Q values. (b) The Q dependence of the XPS spectra in CoFeB­(3 nm)/Pt­(4 nm)/CuO_*x*_(3 nm)/Ta­(2 nm) samples for the Cu 2p transition. XPS spectral intensities were normalized to the Cu 2p_3_/_2_ peak.

The impact of oxidation level on SOT efficiency was investigated by extracting the damping-like SOT efficiency ξ_*DL*_ across different Q values. The dimensionless term of damping-like SOT efficiency can be calculated by eq 1.5, with the detailed derivation and calculation procedure provided in Supporting Information 3.

$$\xi_{DL}=\frac{2e}{\hbar }\mu_{0}M_{s}t_{FM}\cdot \left(\frac{H_{DL}}{J}\right)$$ 1.5

The Q dependence of the |ξ_*DL*_| is shown in Figure 4(a). The sample fabricated with Q = 0%, which represents a nonoxidized Cu layer in the CoFeB­(3 nm)/Pt­(4 nm)/Cu­(3 nm)/Ta­(2 nm) structure, exhibits a |ξ_*DL*_| of 0.17. This value is nearly identical to that of the CoFeB­(3 nm)/Pt­(4 nm) control sample. This agreement enhances the reliability of the overall measurement approach, as it confirms that the additional metallic Cu layer has minimal influence on the damping-like SOT efficiency.

**Figure 4:**
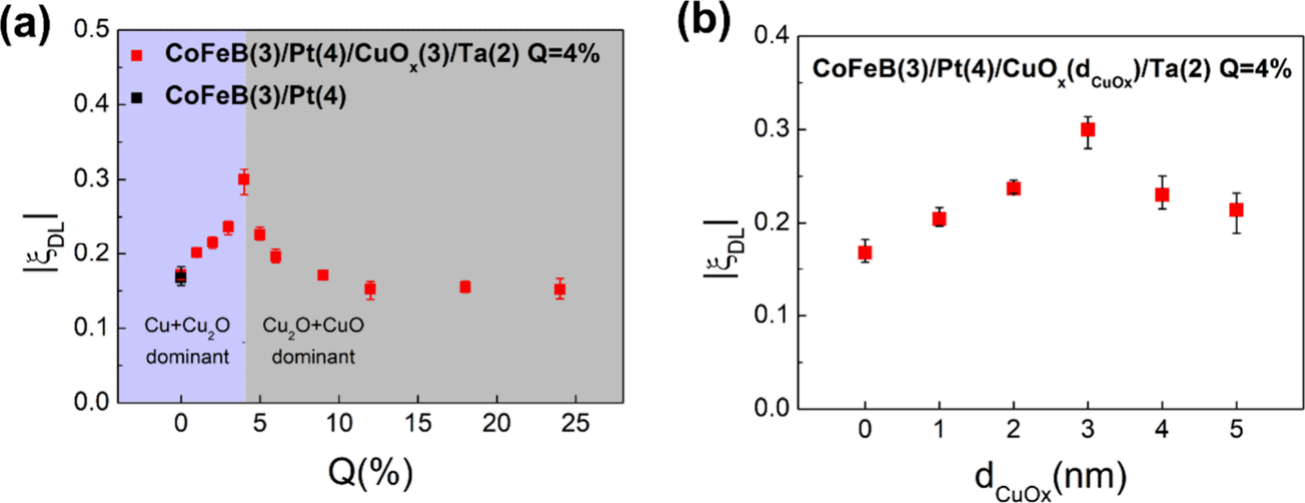
(a) Q dependence of CoFeB­(3 nm)/Pt­(4 nm)/CuO_*x*_(3 nm)/Ta­(2 nm)’s damping-like SOT efficiency. The |ξ_*DL*_| reaches a maximum of 0.30 at Q = 4%. (b) CuO_*x*_ thickness dependence of damping-like SOT efficiency

As Q increases from 0%, |ξ_*DL*_| increases sharply and reaches a maximum of 0.30 at Q = 4%, indicating an approximately 76% enhancement over the CoFeB­(3 nm)/Pt­(4 nm) control sample. However, as Q increases further, |ξ_*DL*_| decreases steadily, returning to a value nearly identical to the control sample by Q = 9%, and becomes slightly suppressed when Q ≥ 12%. Such a decrease in |ξ_*DL*_| is likely related to the reduced current through the CuOx layer, which weakens the role of CuOx in supporting device performance. In addition, if Pt at the Pt/CuOx interface were oxidized under high-Q conditions, the resulting reduction in spin transparency could also suppress|ξ_*DL*_|. A similar peaked trend is also observed when plotting |ξ_*DL*_| against the resistivity of the CuO_*x*_ layer (see Supporting Information 4). This peaked behavior reveals that CuO_*x*_ oxidation plays a decisive role in enhancing or suppressing the damping-like SOT efficiency.

We interpret this behavior as the result of oxidation-driven changes in the electronic structure and the orbital current generation capability of the CuO_*x*_ layer. At low Q, the layer remains largely metallic, and the observed spin–orbit torque is primarily attributed to the spin Hall effect from the adjacent Pt layer. As Q increases and Cu begins to oxidize into Cu_2_O, the film develops a more complex orbital configuration while maintaining moderate conductivity. This intermediate oxidation regime is favorable for orbital current generation, and the enhancement in |ξ_*DL*_| near Q = 4% suggests the activation of orbital transport mechanisms. While the exact mechanism—whether bulk orbital Hall effect (OHE) or interfacial orbital Rashba–Edelstein effect (OREE)—remains under debate, the observed trends point toward a dominant role of orbital angular momentum contributions originating from the Cu_2_O-rich CuO_*x*_ layer. Cu_2_O is reported to retain strong orbital character and is sufficiently conductive to support transverse orbital current flow, which can be partially converted to spin accumulation at the Pt interface. This trend is consistent with previous work on reactive-sputtered CuOx, which reported that under low Q conditions, the |ξ_*DL*_| increases with increasing Q, further supporting the role of controlled oxidation in enhancing orbital-to-spin conversion efficiency.^40^

At higher Q values (Q ≥ 6%), the increased presence of Cu^2+^ species and the appearance of satellite peaks in XPS spectra confirm a transition toward CuO—a more insulating phase. In this regime, the reduced carrier mobility and enhanced resistivity likely hinder orbital current generation and transport, thereby diminishing the overall SOT efficiency. Consequently, the torque enhancement declines and eventually approaches that of the control sample. These findings highlight the sensitivity of orbital torque effects to both the chemical phase and transport characteristics of the CuO_*x*_ layer and underscore the importance of precise oxidation control in engineering efficient orbital torque sources.

Together, these results establish that a moderate oxidation level, where the CuO_*x*_ is predominantly Cu_2_O, optimizes the generation and conversion of orbital currents, leading to enhanced spin–orbit torque. In contrast, under-oxidized (metallic Cu) or overoxidized (CuO-rich) samples yield lower SOT efficiencies. This Q-dependent trend reveals the critical role of precise oxidation control in tuning the electronic structure of CuO_*x*_ and optimizing its function as an orbital current source in spintronic devices. While our analysis captures the systematic evolution of CuO_*x*_ through transport and spectroscopic measurements, we acknowledge as a limitation that absolute oxygen quantification was not performed in this work. Future studies employing techniques capable of precise compositional analysis will be highly valuable to complement our findings and further refine the understanding of CuO_*x*_ chemistry in relation to orbital-current generation.

#### CuO_*x*_ Thickness Dependence

To investigate how CuO_*x*_ thickness influences orbital torque generation, we measured the |ξ_*DL*_| as a function of CuO_*x*_ thickness, with the Pt layer fixed at 4 nm and the oxidation level held at Q = 4%. As shown in Figure 4(b), |ξ_*DL*_| increases with increasing *d*_*CuOx*_ and reaches a maximum at 3 nm. Beyond this point, the efficiency declines noticeably. The initial rise in |ξ_*DL*_| suggests that a thicker CuO_*x*_ layer provides a larger volume for orbital Hall current generation, potentially increasing the amount of orbital angular momentum reaching the CuO_*x*_/Pt interface. Since orbital-to-spin conversion is expected to occur near this boundary, an increase in orbital current available at the interface could lead to enhanced spin injection and torque.

However, the observed decrease in |ξ_*DL*_| for *d*_*CuOx*_ > 3 nm implies that additional thickness does not continue to contribute beneficially. One possible explanation is that orbital angular momentum generated deeper within thicker CuO_*x*_ layers may not effectively couple to the Pt interface for conversion into spin current. Alternatively, variations in interfacial structure or stoichiometry at larger CuO_*x*_ thicknesses could reduce the efficiency of orbital-to-spin conversion. At this stage, we do not rule out other factors, and further investigation would be needed to isolate the underlying mechanism.

These findings indicate that while increasing CuO_*x*_ thickness can enhance orbital torque generation up to a point, excessive thickness may lead to reduced interfacial efficiency or structural changes that limit further improvement. A thickness of around 3 nm appears optimal under the present conditions.

#### Pt Thickness Dependence

We next explored the dependence of damping-like SOT efficiency on the thickness of the Pt layer. The extracted values of |ξ_*DL*_| are shown in Figure 5(a). For the CoFeB­(3 nm)/Pt­(*d*_*Pt*_) control samples, the SOT efficiency exhibits the expected trend predicted by the drift-diffusion model:^41,42^

$$\left|\xi_{DL}\left(d_{HM}\right)\right|=\left|\xi_{DL}^{0}\right|(1-\mathrm{sech}\left(\frac{d_{HM}}{\lambda_{sd}}\right)$$ 1.6

where *d*_*HM*_ is the heavy metal thickness, λ_*sd*_ is the spin diffusion length, and ξ_*DL*_^0^ is the bulk damping-like SOT efficiency. The SOT efficiency increases with Pt thickness and gradually saturates. The spin diffusion length extracted from the CoFeB(3)/Pt­(*d*_*Pt*_) control sample series is approximately 1.2 nm, which is similar to numerous previous works.^34,43,44^

**Figure 5:**
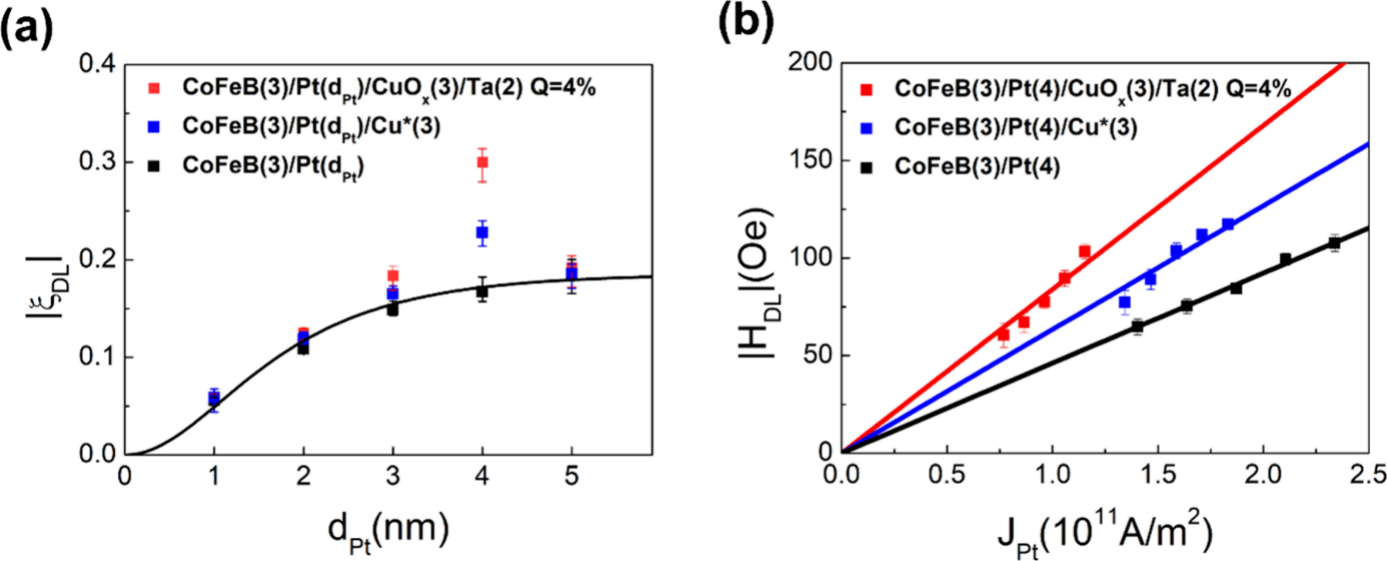
Comparison of CoFeB­(3 nm)/Pt­(4 nm) control sample, CoFeB­(3 nm)/Pt­(4 nm)/Cu*­(3 nm) sample with naturally oxidized, and CoFeB­(3 nm)/Pt­(4 nm)/CuO_*x*_(3 nm)/Ta­(2 nm) sample fabricated via reactive sputtering at Q = 4%. (a) Pt thickness dependence of damping-like SOT efficiency. (b) *H*_*DL*_/*J* comparison.

In contrast, the CoFeB­(3 nm)/Pt­(*d*_*Pt*_)/CuO_*x*_(3 nm)/Ta­(2 nm) with Q = 4% samples exhibit a more nuanced behavior. At 1 nm Pt thickness, the SOT efficiency shows little to no enhancement compared to the control, suggesting that a minimum Pt thickness is required to support efficient orbital-to-spin conversion. As *d*_*Pt*_ increases, |ξ_*DL*_| rises sharply, peaking near 4 nm. This significant enhancement indicates that the orbital current generated in CuO_*x*_ is successfully converted into spin current and transported through Pt to exert torque on the ferromagnet. However, for *d*_*Pt*_ > 4 nm, the enhancement diminishes and |ξ_*DL*_| in CuO_*x*_-inserted samples approaches that of the control. This Pt thickness dependence behavior is also observed in other previous studies.^32,45^

This decline may be attributed to the finite spin diffusion length of Pt. Since the orbital-to-spin conversion is believed to occur primarily near the CuO_*x*_/Pt interface, as Pt becomes thicker, spins converted in that region must traverse the full Pt layer thickness to reach the ferromagnet. As Pt thickness increases, spin relaxation increases, reducing the amount of spin angular momentum that reaches the CoFeB layer and thus weakening torque efficiency. Therefore, additional Pt thickness contributes little to torque and reduces the relative benefit of the CuO_*x*_ layer.

To further assess the role of CuO_*x*_, we compared these results to samples incorporating a naturally oxidized Cu layer instead of a reactively sputtered CuO_*x*_ layer. The naturally oxidized Cu series was prepared using the optimized conditions identified in this work (see Supporting Information 5). As shown in Figure 5(a), the Pt thickness dependence in the naturally oxidized series mirrors the trend observed in the reactively sputtered CuO_*x*_ series, but with reduced magnitude. Little enhancement is seen below 2 nm Pt thickness, while a moderate improvement appears around 4 nm, where the SOT efficiency reaches its peak at |ξ_*DL*_| ≈ 0.23. Beyond 5 nm, |ξ_*DL*_| converges with the control sample. This behavior supports the idea that orbital current generation via simple air exposure of Cu can also enhance SOT, though less effectively than controlled reactive sputtering.

The extracted *H*_*DL*_/*J* ratios are summarized in Figure 5(b). It reveals a clear and consistent ranking across the sample series with optimized results. The device incorporating reactively sputtered CuO_*x*_ (Q = 4%) exhibits the highest damping-like effective field per unit current density—approximately 76% higher than the Pt-only control sample. In comparison, the naturally oxidized Cu sample shows a more moderate enhancement of ∼ 35%. This distinction directly reflects the impact of the oxidation technique on orbital torque generation. While both methods can generate orbital currents and contribute to spin–orbit torque enhancement, reactive sputtering provides superior control over film stoichiometry and thickness uniformity, leading to more effective and reproducible torque delivery.

These results further reinforce the critical role of oxidation level control. Although natural oxidation offers a simpler fabrication route, its lack of tunability limits the extent and consistency of the orbital torque it can generate. In contrast, reactive sputtering enables fine control over CuO_*x*_ properties, allowing the orbital current source to be more efficiently coupled with the spin transport layer. When combined with Pt of optimized thickness, this strategy results in the most pronounced enhancement of damping-like spin–orbit torque.

## Conclusions

In this study, we systematically investigated the enhancement of damping-like spin–orbit torque (SOT) in CoFeB/Pt heterostructures by introducing CuO_*x*_ layers as orbital current sources. Using reactive sputtering to precisely control the oxidation level, we identified an optimal condition (Q = 4%) under which the CoFeB­(3 nm)/Pt­(4 nm)/CuO_*x*_(3 nm)/Ta­(2 nm) structure achieved a torque efficiency of |ξ_*DL*_| ≈ 0.30, representing a ∼ 76% enhancement over the CoFeB­(3 nm)/Pt­(4 nm) control sample. This improvement is attributed to efficient orbital-to-spin conversion near the CuO_*x*_/Pt interface and shows a clear thickness dependence, with the torque efficiency peaking at a CuO_*x*_ thickness of 3 nm. Pt thickness tuning further revealed that the conversion process is most effective at ∼ 4 nm, highlighting the importance of interface and layer engineering in optimizing orbital torque generation.

In contrast, the best-performing naturally oxidized sample exhibited a |ξ_*DL*_| of ∼ 0.23, representing a more modest 35% enhancement compared to the control, with limited tunability. The similarity in Pt thickness dependence between the naturally oxidized and reactively sputtered CuO_*x*_ samples supports the common underlying mechanism of orbital-driven torque. While natural oxidation offers process simplicity, its variability and limited tunability lead to smaller and less consistent enhancements. In conclusion, this work establishes reactively sputtered CuO_*x*_ as a robust, tunable source of orbital torque in metallic heterostructures, offering a scalable pathway for enhancing spin–orbit interactions beyond conventional spin Hall systems. By elucidating the structural and interfacial conditions required for efficient orbital-to-spin conversion, these findings offer fundamental insights into orbital current engineering and practical design strategies for next-generation spintronic systems.

## Supplementary Material



## Data Availability

The data that support the findings of this study are available within the article.

## Notes

Influence of Q value on CuOx sputtering rate (Supporting Information 1), Effect of Ta the capping layer (Supporting Information 2), Definition and calculation of SOT efficiency (Supporting Information 3), Oxidation level and correlation with SOT efficiency (Supporting Information 4), and Effect of natural oxidation time on SOT efficiency (Supporting Information 5) (PDF)
