# Supplementary material for: Oxidation-Tuned CuO x for Spin–Orbit Torque Efficiency Enhancement
Source: ACS Appl Mater Interfaces. 2025 Oct 21;17(44):60845–51. doi: 10.1021/acsami.5c15854 (PMC12598702; doi:10.1021/acsami.5c15854)
Supplement: Supplementary file 1 [file am5c15854_si_001.pdf]

Supporting Information

Oxidation-Tuned CuO_x for Spin-Orbit Torque Efficiency Enhancement

Che-Jui Li[1], and Chi-Feng Pai[1*]

[1]*Department of Materials Science and Engineering, National Taiwan University, Taipei 10617, Taiwan*

To whom correspondence should be addressed: pai.chifeng@gmail.com

Supporting Information 1 | Influence of Q value on CuO_x sputtering rate

Figure S1 shows the sputtering rate of CuO_x thin films as a function of Q. The measured rates at Q = 0%, 6%, 12%, 18%, and 24% are 9.04, 9.15, 9.50, 10.08, and 11.12 s/nm, respectively. At low Q values (0-12%), the sputtering rate remains nearly unchanged. When Q exceeds 12%, the sputtering rate clearly increases in s/nm, which corresponds to a decrease in the actual deposition rate. This result indicates that higher oxygen partial pressure slows down the thin film growth rate.

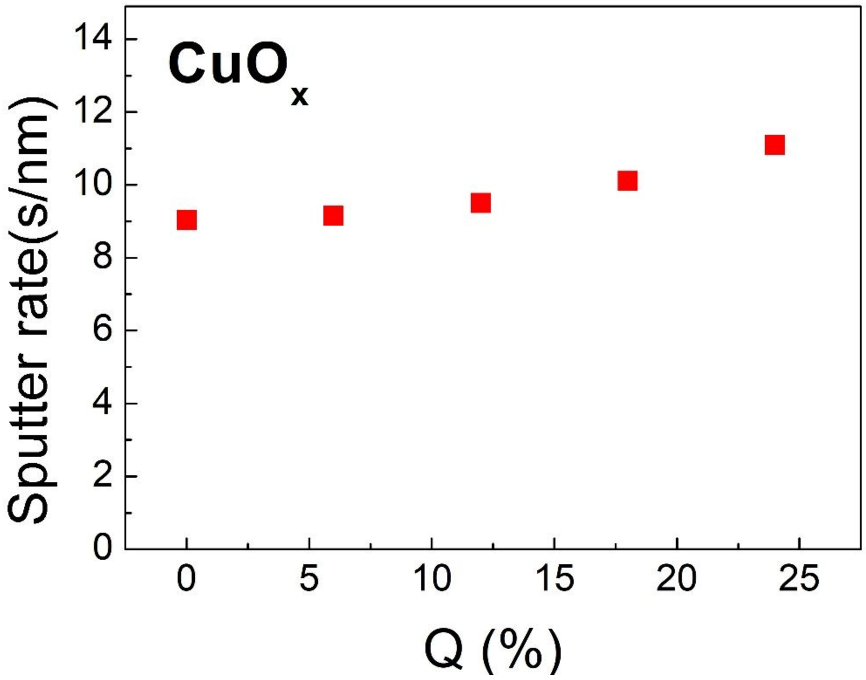

Figure S1 Sputtering rate of CuO_x thin films versus Q values.

Supporting Information 2 | Effect of Ta Capping Layer

To evaluate whether the Ta capping layer influences the extracted ξ_{DL}, control samples of CoFeB(3 nm)/Pt(4 nm)/Ta(d_{Ta}) were prepared with different Ta thicknesses (d_{Ta}= 0, 1, and 2 nm). Since ultrathin Ta capping layers are expected to rapidly oxidize into an insulating TaO_x layer under ambient conditions, no significant effect on spin transport is anticipated. The measured $|\xi_{DL}|$ for the three cases are 0.1673, 0.1619, and 0.1728, respectively, showing negligible variation within experimental error, as summarized in Figure S2. These results confirm that the presence or absence of a thin Ta capping layer does not affect the main conclusions of this work.

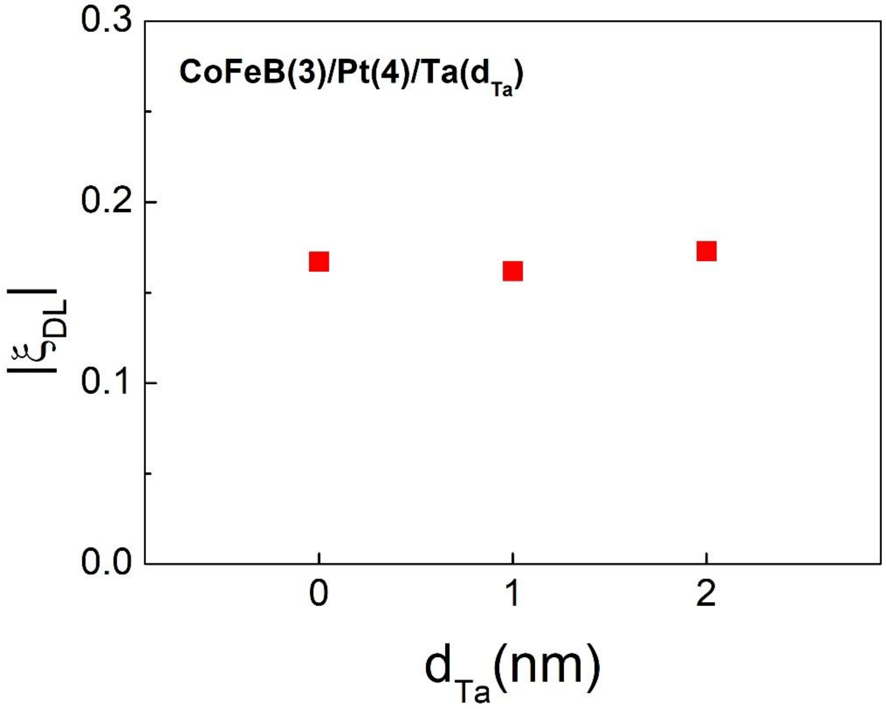

Figure S2 $|\xi_{DL}|$ of CoFeB(3 nm)/Pt(4 nm)/Ta(d_{Ta}) with different Ta capping thicknesses (0, 1, and 2 nm). The $|\xi_{DL}|$ remain nearly unchanged, confirming that the Ta capping layer, which readily oxidizes into TaO_x under ambient conditions, does not influence the experimental results.

Supporting Information 3 | Definition and Calculation of SOT efficiency

The dimensionless term of damping-like SOT efficiency can be defined as

$$\xi_{DL} = \frac{2e}{\hbar}\mu_0 M_s t_{FM} \cdot \left(\frac{H_{DL}}{J}\right) \tag{1}$$

where J denotes the charge current density flowing in the spin source layer. In this study, the multilayer structure under investigation is CoFeB/Pt/CuO_x/Ta, where Pt serves as the spin source layer. Therefore, J specifically corresponds to the current density in Pt. Because the applied current distributes among all conductive layers, the current density in Pt can be written as

$$J_{Pt} = \left(\frac{t_{Pt}/\rho_{Pt}}{t_{Pt}/\rho_{Pt} + t_{CoFeB}/\rho_{CoFeB} + t_{CuOx}/\rho_{CuOx}}\right) \cdot \frac{I_{applied}}{w \cdot t_{Pt}} \tag{2}$$

where w is the device width. Substituting this into the definition of ξ_{DL} yields a final expression directly in terms of the applied current:

$$\xi_{DL} = \frac{2e}{\hbar}\mu_0 M_s t_{CoFeB} w t_{Pt} \cdot \left(\frac{t_{Pt}/\rho_{Pt} + t_{CoFeB}/\rho_{CoFeB} + t_{CuOx}/\rho_{CuOx}}{t_{Pt}/\rho_{Pt}}\right) \cdot \left(\frac{H_{DL}}{I_{applied}}\right) \tag{3}$$

Thus, all values of ξ_{DL} reported in this manuscript are current-density–normalized to the current flowing in Pt. The calculation explicitly incorporates the current shunting effect among Pt, CuO_x, and CoFeB layers. The Ta capping layer rapidly oxidizes into an insulating TaO_x layer under ambient conditions. Therefore, Ta does not contribute to charge transport or spin–orbit torque generation and is excluded from the current shunting calculation.

In addition to the current-density–normalized definition used in this work, the damping-like SOT efficiency can also be expressed in an electric field-normalized form:

$$\xi_{DL}^{E} = \frac{2e}{\hbar}\mu_0 M_s t_{FM} \cdot \left(\frac{H_{DL}}{E}\right) \tag{4}$$

where E is the applied electric field across the multilayer structure. Since all conductive layers are connected in parallel, they experience the same electric field. The relation between J_{Pt} and E is simply Ohm's law:

$$E = J_{Pt} \cdot \rho_{Pt} \tag{5}$$

Substituting this into the definition of ξ_{DL} leads to the direct conversion between the two definitions:

$$\xi_{DL}^{E} = \xi_{DL}/\rho_{Pt} \tag{6}$$

Therefore, the current-density–normalized and field-normalized definitions differ only by the scaling factor given by the Pt resistivity. In this manuscript, all main-text data are presented in the current-density–normalized form ξ_{DL}, which explicitly accounts for current shunting into Pt. For completeness, we provide in the Supporting Information the field-normalized values ξ_{DL}^{E} as a function of Q (Figure S3). The overall trend remains identical, with only a rescaling in magnitude.

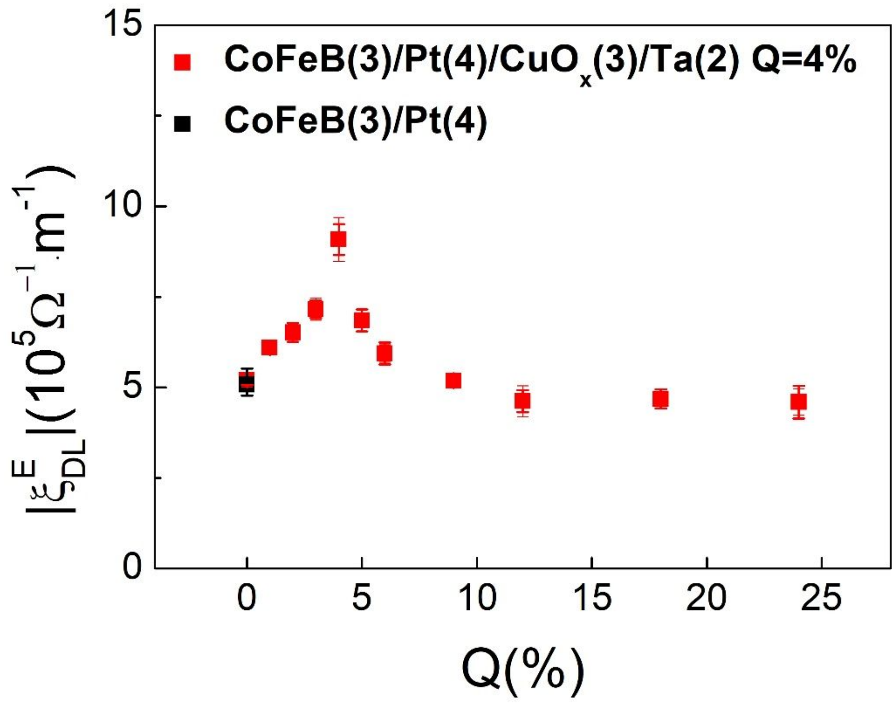

Figure S3 Field-normalized damping-like SOT efficiency ξ_{DL}^{E} as a function of oxygen flow ratio Q for CoFeB(3 nm)/Pt(4 nm)/CuO_x(3 nm)/Ta(2 nm) and the CoFeB(3 nm)/Pt(4 nm) control sample. The overall trend is consistent with the current-density–normalized data.

Supporting Information 4 | Oxidation Level and Correlation with SOT Efficiency

The correlation between the resistivity of CuO_x and the damping-like SOT efficiency was examined, as shown in Figure S4. $|\xi_{DL}|$ increases with resistivity at first, reaches a maximum around 20 μΩ·cm, and decreases when the resistivity becomes larger. This trend is consistent with the dependence on Q value observed in this study.

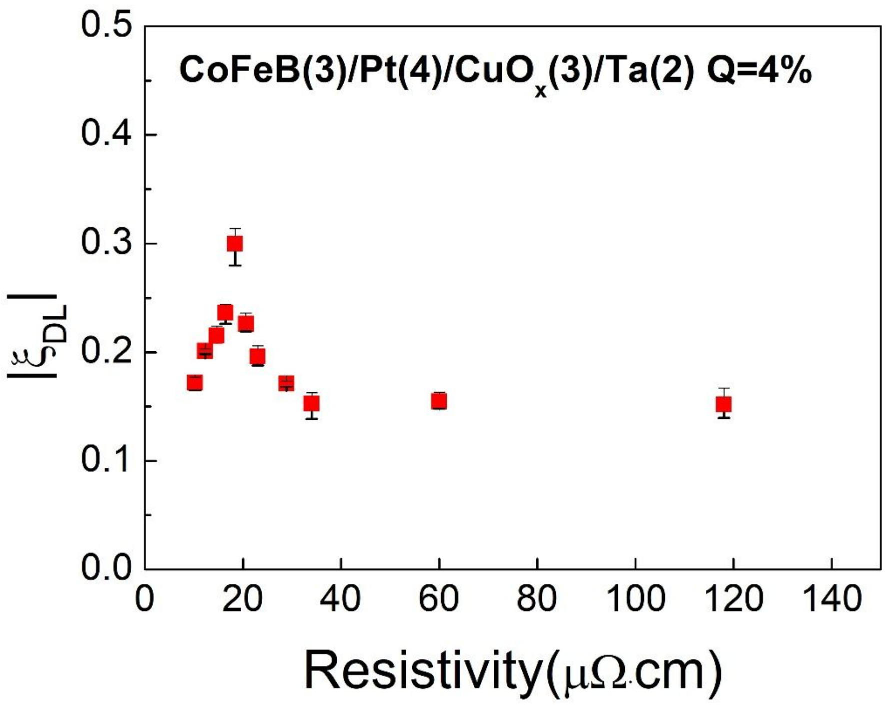

Figure S4 $|\xi_{DL}|$ of CoFeB(3 nm)/Pt(4 nm)/CuO_x(3 nm)/Ta(2 nm) (Q = 4%) as a function of CuO_x resistivity.

Supporting Information 5 | Effect of natural oxidation time on SOT efficiency

To study the influence of natural oxidation, CoFeB(3 nm)/Pt(4 nm)/Cu*(3 nm) samples, where Cu* denotes a naturally oxidized Cu layer, were exposed to air for different durations before measurement. As shown in Figure S5, the $|\xi_{DL}|$ increases during the first day of oxidation, but then decreases with further exposure. After three days, the $|\xi_{DL}|$ is reduced to a level comparable to that of the CoFeB(3 nm)/Pt(4 nm) control sample. These results indicate that while initial oxidation of the Cu layer can enhance the $|\xi_{DL}|$, excessive natural oxidation reduces it, ultimately diminishing the beneficial effect.

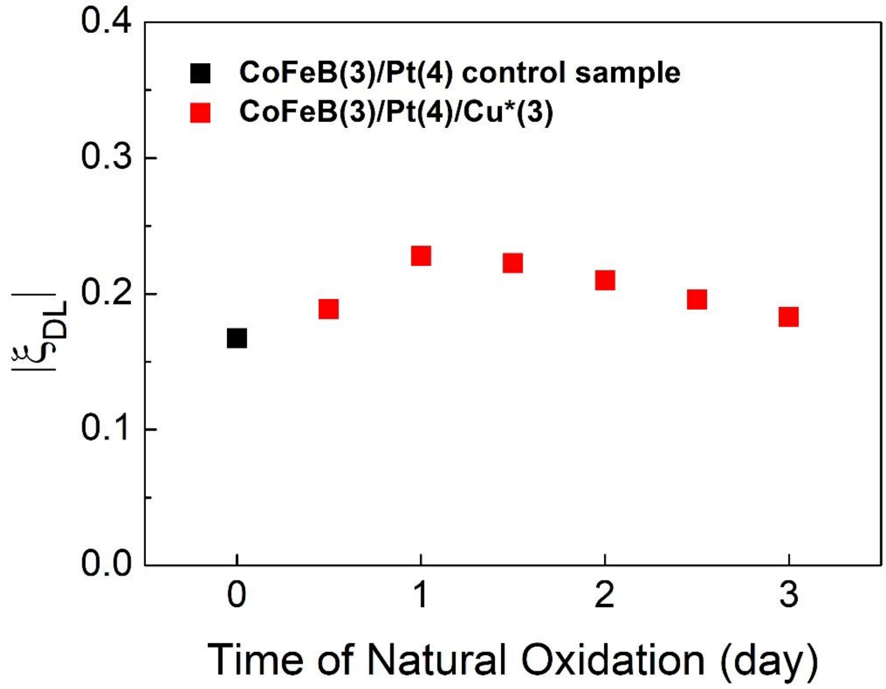

Figure S5 $|\xi_{DL}|$ as a function of natural oxidation time for CoFeB(3 nm)/Pt(4 nm)/Cu*(3 nm) heterostructures. The $|\xi_{DL}|$ peaks after one day of oxidation but decreases upon extended exposure, with excessive oxidation lowering the $|\xi_{DL}|$ to the level of the CoFeB(3 nm)/Pt(4 nm) control sample.
